# Assessment of different phytogenic-based additives on *in vitro* rumen fermentation profile and methane emissions

**DOI:** 10.3389/fvets.2025.1591700

**Published:** 2025-05-01

**Authors:** Hani M. El-Zaiat, Hussein A. Masood, Samira S. Al Hinai, Reem H. Al Maamari, Sanad S. Al Riyami, Kaadhia Al-Kharousi, Azza H. Al-Salami, Nasser Al-Habsi

**Affiliations:** ^1^Department of Animal and Veterinary Sciences, College of Agricultural and Marine Sciences, Sultan Qaboos University, Muscat, Oman; ^2^Department of Food Science and Nutrition, College of Agricultural and Marine Sciences, Sultan Qaboos University, Muscat, Oman

**Keywords:** feed degradability, methane mitigation, phenolic acids, ruminal characteristics, tropical additives

## Abstract

**Introduction:**

Phytogenic feed additives have gained increasing attention in ruminant nutrition due to their capacity to modulate ruminal fermentation and reduce methane (CH_4_) emissions. This study evaluated the effects of three plant-based additives.

**Methods:**

Neem leaf (*Azadirachta indica*; NL), Indigofera leaf (*Indigofera oblongifolia*; IL), and Pumpkin peel (*Cucurbita pepo*; PP) included at four levels (0, 10, 20, and 30 g/kg DM) on *in vitro* ruminal fermentation, feed degradability, and CH_4_ emissions. A total mixed ration (TMR) was incubated with each additive and buffered rumen fluid using the *in vitro* gas production technique for 24 hours.

**Results and discussion:**

NL and IL supplementation significantly (*p* < 0.05) increased gas and carbon dioxide (CO_2_) production, organic matter degradability, and total volatile fatty acid concentrations, particularly propionate and butyrate. Simultaneously, acetate concentration, CH_4_ emissions, NH_3_-N levels, and protozoa abundance were reduced (*p* < 0.05). However, PP had limited effects on these parameters. The phenolic profiles of NL and IL, notably rich in 2-hydroxycinnamic acid and p-coumaric acid, likely contributed to these outcomes. In conclusion, these findings support the use of NL and IL as effective phytogenic additives for improving rumen fermentation and mitigating CH_4_ production. Further *in vivo* trials are recommended to validate these *in vitro* results.

## Introduction

The use of locally available phytogenic feed additives is increasingly recognized as a sustainable approach to improve ruminant nutrition and reduce environmental impacts. Plant-derived bioactive compounds, particularly phenolics and flavonoids, have been widely documented for their capacity to modulate ruminal fermentation, enhance nutrient utilization, and decrease methane (CH₄) emissions (1–3).

Among these, *Indigofera oblongifolia* and *Azadirachta indica* (neem) are multipurpose plants that thrive under arid and semi-arid conditions. Their leaves are rich in phenolic acids and flavonoids substances, which have shown antimicrobial and anti-inflammatory properties with potential effects on rumen microbial ecology (4–6). Indigofera is a wild multi-use leguminous shrub in the *Fabaceae* family that is widely distributed in Africa, South Asia and Southeast Asia (6). Most of the species of Indigofera generally grow in different areas as well as withstand extremes of drought and temperature environmental conditions. However, neem plant belongs to the family *Meliceae* and has been used to modulate rumen ecology due to the modulatory potentials of its secondary bioactive (7). Besides, Pumpkin (*Cucurbita pepo*) belongs to the family *Cucurbitaceae* and is used as remedies, pharmaceutics, and functional foods sectors for their extreme chemical properties and biological activities (8). Pumpkin peel, an agro-industrial by-product, is also rich in antioxidant and bioactive phytonutrients including carotenoids, tocopherols, and polyphenols (8, 9). Our previous study has demonstrated that dietary inclusion with 40 g of neem leaf powder can modulate rumen fermentation, enhance fiber digestibility, and improve growth performance in small ruminants (10).

However, comprehensive comparative studies evaluating these phytogenic sources at graded inclusion levels under *in vitro* conditions on rumen fermentative activity and CH_4_ emissions are still lacking. Therefore, the objective of this study was to assess the effects of incremental inclusion levels (0, 10, 20, and 30 g/kg DM) of neem leaf, indigofera leaf, and pumpkin peel powders on *in vitro* rumen fermentation characteristics, feed degradability, and CH_4_ emissions. We hypothesized that phytogenic additives, particularly those with higher phenolic acid content, would improve fermentation efficiency and mitigate CH₄ production.

## Materials and methods

### Study location and ethics

This study was conducted at the Feed Analysis and Food Science Laboratories, College of Agricultural and Marine Sciences (CAMS), Sultan Qaboos University (SQU), Muscat, Oman.

### Phytogenic plants plant material collection and processing

Neem, Indigofera, and pumpkin plants were harvested from the university’s experimental field station (23°59′89.8″ N, 58°16′25.6″ E). Leaves of neem and Indigofera were air-dried and manually chopped. However, pumpkin peels were washed, separated, air-dried, and ground into a fine powder using a laboratory blender.

### Phenolic compound analysis

For phenolic acid analysis, 1.0 g of each powdered plant material was extracted with 10 ml of analytical-grade methanol in a centrifuge tube. Samples were vortexed, sonicated at 25°C for 30 min, and filtered through a nylon syringe filter. Phenolic acids were quantified using ultra-performance liquid chromatography (UPLC) equipped with a Shimadzu SIL-30A autosampler (Tokyo, Japan) according to the method descripted by El Hilaly et al. (11) with some modifications.

### Experimental diet and treatments

The total mixed ration (TMR; 50:50 forage to concentrate) consisted of Rhodes grass (*Chloris gayana*) hay (50%), ground yellow corn grain (27%), soybean meal (11.50%), wheat bran (10.30%), calcium carbonate (0.70%), sodium chloride (0.25%) and mineral and vitamin mixture (0.25% containing: 1.00% Ca; 0.60% P; 0.3 mg/kg Se; 8,800 IU/kg of vitamin A,; 2,200 IU/kg of vitamin D, and 33 IU/kg of vitamin E). The chemical composition of the TMR was determined according to AOAC (12) procedure for dry matter (DM) by drying in air-forced oven at 65°C to a constant weight. Ash content was determined by ashing the sample at 600°C in Muffle furnace for 2 h. Organic matter (OM) was calculated as DM—ash content. The ether extract (EE) was analyzed by Soxhlet extraction procedure. Total nitrogen (TN) was measured using a nitrogen Analyzer (BUCHI KjelMaster K-375, Flawil, Switzerland), and crude protein (CP) content was calculated as 6.25 × TN according to Kjeldahl procedure. Fiber fractions (NDF and ADF) were determined (13, 14). The content of non-fiber carbohydrates (NFC, g/kg) were calculated. The TMR chemical composition on DM basis was: 89.92% DM, 9.31% Ash, 12.86% CP, 2.28% EE, 46.74% NDF, 26.27% ADF, and 28.83% NFC.

The TMR was ground and supplemented with NL, IL, or PP at 0, 10, 20, or 30 g/kg DM. Each treatment was replicated six times in a completely randomized design.

### *In vitro* gas production

This *in vitro* study utilized the GP technique using 100-ml glass syringes fitted with plungers in different incubation intervals following the methods described by Menke and Steingass (15). Briefly, a 0.500 g of each basal TMR (DM basis) along with its respective additive level, was placed into 100 ml glass syringes fitted with plungers. Rumen fluid was obtained from a fistulated Holstein cow (450 ± 15 kg BW) before morning feeding. The rumen contents were filtered and maintained at 39°C. The rumen-buffer solution was prepared according to the method described by Menke and Steingass (15). Each syringe was injected with 30 ml of buffered rumen fluid (10 ml rumen fluid: 20 ml buffer). The syringes were purged with CO₂ and incubated at 39°C for 24 h. Each phytogenic additive (n = 4 levels/experiment) was tested in six replicates, along with four blank syringes, incubated in three separate runs repeated over two different weeks. Therefore, the total number of syringes each tested additive was: 84 syringes (4 treatment levels × 6 replicates +4 blanks) × 3 runs.

### Total gas, CH_4_ and CO_2_ production measurements

Net gas production (GP) was recorded at 2, 4, 8, 12, and 24 h using the syringe scale by subtracting the blank syringe (containing only rumen fluid and buffer solution) values. At each time point, 1 ml gas samples were collected from the empty area of the syringe for CH_4_ and CO_2_ concentrations analysis via gas chromatography (Agilent, model GC, 6890 N, California, US) a TCD-back detector according to Budiman and Zuas (16). After each sampling time point, the cumulative gas was vented after each reading to maintain microbial activity (17).

### Feed degradability and rumen fermentation attributes measurement

Immediately after 24 h of incubation, the undigested residue in each syringe was transferred into pre-weighed porcelain crucible and dried in an oven at 65°C for 72 h, then assayed for DM and OM contents. The *in vitro* degradability of DM and OM was calculated based on the weight lost after 24 h following the protocol described by Al-Hasani et al. (18).

At 24 h of incubation, the buffered-rumen fluids pH was measured using a digital pH meter (HANNA Instruments, HI Microcomputer 9,025, Smithfield, VA, USA) and rumen fluid was centrifuged. The supernatant was assayed for the concentrations of ammonia nitrogen (NH_3_-N) using the method described by Konitzer and Voigt (19) and volatile fatty acid (VFA) following the procedure described by Al-Hasani et al. (18) and El-Zaiat et al. (20) analyses. The VFA were analyzed using a gas chromatograph (Agilent, model GC, 6890 N, California, US). The individual VFAs were identified using a polyethylene glycol TPA capillary column (HP-FFAP, Agilent 19091F-115) and a flame ionization detector (FID) set at a temperature of 260°C. The hydrogen was used as the carrier gas at a flow rate of 40 ml/min. Total protozoa count was performed according to the procedures of Dehority et al. (21).

### Statistical analysis

All data were analyzed using the GLM procedure of SAS 9.1 software (SAS Inst. Inc., Cary, NC, USA). The linear and quadratic contrasts were applied to evaluate the effect of additive incremental level responses. Duncan’s test was used to assess the significant differences at *p* ≤ 0.05.

## Results

### Phenolic composition of plant additives

Table 1 presents the phenolic acid composition of the three plant-based additives. Indigofera leaf (IL) exhibited the highest total phenolic content (412.56 mg/g DM), followed by neem leaf (NL; 291.68 mg/g DM), and Pumpkin peel (PP; 63.68 mg/g DM). Furthermore, p-Coumaric (145.05 mg/g DM) and ferulic acids (104.65 mg/g DM) were the predominant phenolics in IL, while 2-hydroxycinnamic acid was the major compound in NL. The PP additive exhibited only low concentrations of caffeic acid (17.00 mg/g DM), syringic acid (14.95 mg/g DM), p-coumaric acid (8.75 mg/g DM), and ferulic acid (6.24 mg/g DM).

**Table 1:** Principal identified phenolic acids compounds of the evaluated phytogenic supplements using ultra-performance liquid chromatography.

Peak No.	Compounds	RT (min)	MF	MW(g/mol)	Concentration (mg/g DM)		
					NL	IL	PP
1	Gallic acid	6.61	C_7_H_6_O_5_	170.12	0.00	0.00	0.00
2	3,4-dihydroxybenzoic acid	10.98	C_7_H_6_O_4_	154.12	0.00	0.00	2.90
3	Chlorogenic acid	16.28	C_16_H_18_O_9_	354.30	79.43	104.18	0.00
4	Vanillic acid	17.62	C_8_H_8_O_4_	168.14	25.02	0.00	5.14
5	Caffeic acid	18.51	C_9_H_8_O_4_	180.16	36.09	27.69	17.00
6	Syringic acid	19.98	C_9_H_10_O_5_	198.17	0.00	0.00	14.95
7	p-Coumaric acid	22.88	C_9_H_8_O_3_	164.04	0.00	145.05	8.75
8	Ferulic acid	25.24	C_10_H_10_O_4_	194.18	35.22	135.65	6.24
9	2-Hydroxycinnamic acid	29.16	C_9_H_8_O_3_	164.16	99.04	0.00	0.00
10	Cinnamic acid	34.96	C_9_H_8_O_2_	148.15	16.89	0.00	0.00
Total					291.68	412.56	54.96

RT, retention time; MF, molecular formula; MW, molecular weight; NL, neem leaf; IL, Indigofera leaf; PP, pumpkin peel.

### Total GP, CH_4_ and CO_2_ emission, and feed degradability

As shown in Table 2, net GP after 24 h increased linearly (*p* < 0.05) with rising inclusion levels of NL and IL but not with PP. All NL, IP, and PP levels linearly increased (*p* < 0.05) CO_2_ production. Conversely, CH_4_ production declined significantly (*p* < 0.05) in response to increasing inclusion levels of NL, IL, and PP. However, OM degradability increased linearly with NL and IL (*p* < 0.05), but no significant effect was observed with PP.

**Table 2:** Effect of different phytogenic supplements on *in vitro* gas production (GP), methane production (CH_4_) and substrate degradation within 24 h of incubation.

Items	Treatments	Levels (g/kg DM)				SEM	*p*-values	
		0	10	20	30		Linear	Quadratic
Net GP, ml/kg OM	NL	125	123	133	132	1.70	0.041	0.597
	IL	129	129	130	131	1.63	0.034	0.326
	PP	131	134	131	132	2.94	0.543	0.317
Net CH_4_, ml/kg OM	NL	10.30	8.43	9.41	9.03	0.22	0.028	0.241
	IL	9.65	7.81	7.58	7.12	0.48	0.021	0.531
	PP	11.32	9.19	9.26	9.21	0.91	0.046	0.635
Net CO_2_, ml/kg OM	NL	18.44	18.92	22.47	24.48	1.31	0.039	0.591
	IL	23.05	23.25	24.36	24.61	0.20	0.031	0.691
	PP	23.81	23.08	24.54	26.23	0.34	0.015	0.814
DM degradability, g/kg OM	NL	597	604	603	600	10.10	0.435	0.335
	IL	556	553	551	559	1.19	0.241	0.445
	PP	562	567	558	558	0.76	0.621	0.172
OM degradability, g/kg DM	NL	569	632	657	664	7.64	0.032	0.642
	IL	564	583	587	589	2.02	0.049	0.136
	PP	568	572	570	569	1.18	0.131	0.828

NL, neem leaf; IL, Indigofera leaf; PP, pumpkin peel; GP, gas production; OM, organic matter; DM, dry matter; SEM: means standard error.

### Rumen fluid fermentation end-products characteristics

Table 3 displays the effects of the additives on ruminal pH, NH_3_-N, and protozoa abundance. After 24 h of incubation, ruminal pH was unaffected by additive inclusion (*p* > 0.05). However, ruminal NH_3_-N concentration and protozoal counts decreased linearly (*p* < 0.05) with increasing levels of NL and IL. According to Table 4, total VFA concentration increased linearly with all levels of NL (*p* = 0.047) and IL (*p* = 0.031), while no change was observed with PP. Acetate proportion decreased linearly and propionate and butyrate proportions increased with all three additives (*p* < 0.05), leading to a linear reduction (*p* < 0.05) in acetate-to-propionate ratio.

**Table 3:** Effect of different phytogenic supplements on *in vitro* ruminal pH, ammonia-nitrogen (NH_3_-N) concentration and protozoa abundance within 24 h of incubation.

Items	Treatments	Levels (g/kg DM)				SEM	*p*-values	
		0	10	20	30		Linear	Quadratic
Ruminal pH	NL	6.15	6.32	6.33	6.33	0.04	0.791	0.847
	IL	6.23	6.21	6.22	6.40	0.04	0.521	0.538
	PP	6.22	6.13	6.24	6.33	0.04	0.519	0.612
NH_3_-N, mg/dl	NL	22.58	20.15	19.94	19.59	0.48	0.028	0.632
	IL	22.79	22.75	21.31	21.50	0.30	0.041	0.359
	PP	21.55	21.28	19.32	18.88	0.53	0.039	0.491
Protozoa, ×10^5^ cell/ml	NL	2.15	1.91	1.89	1.73	0.11	0.046	0.813
	IL	3.21	2.83	2.76	2.42	0.09	0.037	0.266
	PP	4.52	3.91	3.42	3.19	0.20	0.018	0.253

NL, neem leaf; IL, Indigofera leaf; PP, pumpkin peel; SEM: means standard error.

**Table 4:** Effect of different phytogenic supplements on *in vitro* ruminal volatile fatty acids (VFA) profile within 24 h of incubation.

Items	Treatments	Levels (g/kg DM)				SEM	*p*-values	
		0	10	20	30		Linear	Quadratic
Total VFA, mM	NL	108	109	111	114	0.56	0.047	0.589
	IL	94.90	95.85	96.81	98.24	0.62	0.031	0.529
	PP	133	131	131	130	0.54	0.437	0.510
Individual VFA, mol/100 mol								
Acetate	NL	63.19	61.58	59.86	58.72	0.85	0.015	0.598
	IL	68.36	65.62	63.52	64.12	0.93	0.042	0.712
	PP	70.12	62.44	62.54	58.23	2.16	0.037	0.371
Propionate	NL	15.33	17.05	17.82	18.66	0.49	0.015	0.569
	IL	16.38	19.21	19.23	18.98	0.60	0.027	0.439
	PP	17.12	19.05	19.75	20.17	0.58	0.021	0.825
Butyrate	NL	9.78	10.70	11.96	13.46	0.37	0.035	0.598
	IL	6.23	6.98	7.56	9.91	0.69	0.049	0.382
	PP	11.04	14.17	13.31	13.49	0.60	0.047	0.271
Branched-VFA	NL	1.47	1.51	1.50	1.55	0.07	0.621	0.762
	IL	0.36	0.33	0.33	0.46	0.04	0.538	0.581
	PP	0.48	0.61	0.42	0.59	0.08	0.318	0.591
Acetate to propionate ratio	NL	4.14	3.63	3.34	3.16	0.12	0.037	0.587
	IL	4.21	3.39	3.30	3.38	0.18	0.022	0.172
	PP	4.11	3.25	3.15	2.91	0.23	0.021	0.280

NL, neem leaf; IL, Indigofera leaf; PP, pumpkin peel; SEM, means standard error.

## Discussion

The *in vitro* short-term fermentation techniques have been thoroughly applied as a rapid and cost-effective assessment approach to investigate the potential of natural-based additives on rumen fermentation patterns and CH_4_ emission (18, 22). This study evaluated the effects of three phytogenic feed additives on *in vitro* rumen fermentation, gas emissions, and nutrient degradability. The results demonstrated that Neem leaf (NL) and Indigofera leaf (IL) significantly influenced fermentation parameters, whereas Pumpkin peel (PP) exhibited limited effects. The current *in vitro* study is the first to evaluate whether NL, IL and PP derived additives can modify the rumen fermentation profile, including methanogenesis suppression. All phytogenic-based feed ingredients used had effects on ruminal fermentation parameters that were measured. Consistent with previous findings (23, 24), our current results showed that p-coumaric acid and ferulic acid present in IL additive effectively modified ruminal fermentation characteristics by affecting both rumen microbiota and/or their enzyme activity. Additionally, 2-Hydroxycinnamic acid, one of major phenolic acid metabolites represents 33.90% of NL (99.04 mg/g DM) compounds. This compound may potentially affect the enzyme activity of rumen microbes due to their antimicrobial and anti-inflammatory properties (5). It has been well documented that greater substrate degradability demonstrated higher GP (25). In this context, increased net GP in this study coupled with greater substrate degradation rate (22, 26). The predominance of phenolic acids profile in the tested additives substantially affected TMR digestion and fermentability, resulted in increased OM degradability through maintaining the ruminal pH in normal ranges after 24 h of incubation. The increased gas and CO₂ production observed with NL and IL inclusion are indicative of enhanced microbial fermentation, likely driven by the abundance of phenolic compounds such as p-coumaric and 2-hydroxycinnamic acids. These compounds have been shown to modulate microbial populations and enzymatic activity in the rumen (5, 23). The improvement in organic matter degradability with NL and IL further supports the hypothesis that these bioactives enhance ruminal fermentability. A notable finding was the consistent reduction in CH₄ production across all additive levels for NL and IL (14 and 35%, respectively). This supports earlier evidence that the phenolic compounds can disrupt methanogenesis by altering microbial hydrogen use, favoring propionate formation over acetate (27, 28). These mode of action against the rumen microorganisms involves damaging the cell membranes and structures and also by the inactivation of cell enzymes (29). Comparable findings were reported by Haisan et al. (30), greater propionate production at the expense of acetate could be responsible for the greater CO_2_ production in the rumen.

The reduced acetate-to-propionate ratio observed aligns with this mechanism, indicating a shift towards glucogenic fermentation pathways that are energetically more favorable and environmentally beneficial. Therefore, the reduced fiber digestion further reveals the antimicrobial effects of the phenolic metabolites of the additives tested on the rumen microbiome as evidenced by reduced acetate production. These findings suggest that differences in the potentials of these phytonutrients (phenolic compounds) could be the result of differences in the bacterial (Gram-negative and Gram-positive) species cell wall structures (29). These findings have suggested that feed ingredients used could be considered feed additive candidates for use in ruminants. The ruminal VFAs produced are considered the primary source of metabolizable energy supply for ruminants (31). Although PP supplementation decreased acetate and increased propionate concentrations, it did not significantly affect gas production or total VFA concentration. This could be attributed to its relatively low phenolic content, as confirmed by UPLC analysis. These findings suggest that the effectiveness of phytogenic additives depends not only on their botanical source but also on their phytochemical composition and concentration. The decline in NH₃-N concentrations and protozoa abundance with NL and IL addition points to improved nitrogen utilization and possible anti-protozoal effects of their bioactives. Such effects may contribute to reduced protein degradation in the rumen, aligning with the observed decrease in ammonia levels. This also has implications for reducing nitrogen excretion and its associated environmental impact (32).

Increased propionate production is closely associated with reduced methanogenesis in the rumen (28). Additionally, propionate formation mostly competes with the methanogenesis for the hydrogen available in the rumen (33). A consistent finding when P-Coumaric acid was included in the *in vitro* fermentation is the inhibition of *Ruminococcus albus* and *Ruminococcus flavefaciens* in the rumen, leading to reduce fiber degradation (24). Thus, altered ruminal VFA profile might be related to the presence of phytonutrient phenolic used on ruminal microflora, shifting the microbiota activities away from acetate through diverted hydrogen to more propionate, and subsequently hence greater CH_4_ suppression (37). While promising, the current findings are limited by the *in vitro* nature of the study, which does not capture the full complexity of rumen dynamics *in vivo*. Moreover, microbial profiling was not conducted, which would have strengthened the understanding of the mechanisms underpinning the observed responses. Future studies should explore microbial population shifts and validate these results under practical feeding conditions. Additional influences from the presence of phenolic compounds in tested additives that may protect the dietary protein from ruminal degradation (34), resulting in large NH_3_-N concentration reduction reported herein. Consequently, reduced N losses may be expected through feces and urine excretion, taking into account mitigating the environmental impact.

Interestingly, the ruminal methanogenesis is strongly influenced without adversely affecting the other ruminal fermentation parameters, such as the VFA profile. The reason for the small reduction in CH_4_ production might be that ruminal microbes susceptibility could be adapted against the presence of flavonoids and phenolic metabolites (35). As a result, the effects on ruminal acetogens and methanogens could be expected, which may explain the pronounced reduction in acetate and CH_4_ production as a result of reduced fiber degradation. However, the phenolic compounds exhibit antimicrobial properties by reducing ruminal protozoal abundance (36), which may be another reason for the decreased CH_4_ formation (35). Thus, the linear reduction in CH_4_ formation is likely not to be solely ascribed to the changes in the VFAs profile. The present study highlights the potential of NL and IL as effective phytogenic additives to enhance rumen fermentation and mitigate methane and nitrogen emissions. Their rich phenolic content appears to be central to these effects. Although PP contains some bioactive compounds, it demonstrated only modest benefits in this context.

## Conclusion

This *in vitro* study demonstrated that neem and indigofera leaf powders, when included at increasing dietary levels, can positively influence ruminal fermentation dynamics by enhancing gas production, organic matter degradability, and volatile fatty acid profiles, while concurrently reducing methane and ammonia nitrogen concentrations. These effects are likely attributable to their high phenolic acid content. In contrast, pumpkin peel powder exhibited minimal influence on fermentation parameters, potentially due to its lower phytochemical content. These findings support the potential use of neem and indigofera leaf powders as natural phytogenic additives to promote environmentally sustainable ruminant feeding strategies. However, further *in vivo* research is necessary to confirm their efficacy under practical animal production conditions.

## Data Availability

The raw data supporting the conclusions of this article will be made available by the authors, without undue reservation.
